# Assessment of Ondansetron-Associated Hypokalemia in Pediatric Oncology Patients

**DOI:** 10.5402/2012/798239

**Published:** 2012-09-19

**Authors:** Elsa Fiedrich, Vikram Sabhaney, Justin Lui, Maury Pinsk

**Affiliations:** Division of Pediatric Nephrology, University of Alberta, 11405-87 Avenue, Edmonton, AB, Canada T6G 1C9

## Abstract

*Objectives*. Ondansetron is a 5-hydroxytryptamine (5-HT_3_, serotonin) receptor antagonist used as antiemetic prophylaxis preceding chemotherapy administration. Hypokalemia is a rare complication of ondansetron, which may be underreported due to confounding emesis and chemotherapy-induced tubulopathy. We performed a prospective cohort study to determine if ondansetron caused significant hypokalemia independently as a result of renal potassium wasting. *Methods*. Twelve patients were recruited, with ten completing the study. Blood and urine samples were collected before and after ondansetron administration in patients admitted for intravenous (IV) hydration and chemotherapy. Dietary histories and IV records were analyzed to calculate sodium and potassium balances. *Results*. We observed an expected drop in urine osmolality, an increase in urine sodium, but no statistically significant change in sodium or potassium balance before and after ondansetron. *Conclusion*. Ondansetron does not cause significant potassium wasting in appropriately hydrated and nutritionally replete patients. Careful monitoring of serum potassium is recommended in patients with chronic nutritional or volume status deficiencies receiving this medication.

## 1. Introduction

Ondansetron is an antiemetic used as an adjunctive to chemotherapy in pediatric oncology patients. It is a selective 5-HT_3_ receptor antagonist which has an excellent safety profile and is efficacious [1, 2]. A side effect infrequently reported is the development of hypokalemia [3]. Hypokalemia may be a result of ondansetron itself [3–5], an effect of chemotherapy on renal tubular function [6], a result of emesis-induced alkalosis [7], or a combination of factors. 

Based on in vitro data, ondansetron has the capacity to cause hypokalemia by affecting renal tubular physiology [4]. Ondansetron acts at two levels in the nephron. First, at the level of the Loop of Henle, ondansetron downregulates the Na^+^-K^+^-2Cl-(NKCC2) cotransporter, which results in increased sodium delivery to the distal nephron. This in turn necessitates K^+^ excretion, via the ROMK potassium channel to facilitate the electroneutral reabsorption of sodium via the epithelial sodium channel (ENaC) from the distal nephron, leading to K^+^ wasting. Second, throughout the nephron, and in particular the distal tubule, ondansetron upregulates the Na^+^-K^+^ ATPase. This exacerbates the renal K wasting by lowering intracellular sodium levels in distal tubular cells expressing ENaC thereby further increasing tubular sodium entry at this segment. This ultimately requires increased K secretion into the urine via ROMK to maintain electroneutrality. Based on in vitro data, this effect on the renal tubules appears specific to ondansetron and is not a characteristic of other selective 5-HT_3_ antagonists [8].

We previously described an association between ondansetron use and the reproducible development of hypokalemia in a pediatric oncology patient [5]. On review, ondansetron was recognized as a potential causative agent. Within 24 hours of discontinuation of ondansetron, the patient's potassium level and transtubular potassium gradient (TTKG) returned to acceptable levels. The patient was readmitted one month later for IV cyclophosphamide therapy, and a single dose of ondansetron was administered as standard anti-emetic prophylaxis. The TTKG rose to an inappropriately high level has given the patient's volume status, with a concurrent drop in the patient's plasma potassium level.

In that case report, we questioned whether hypokalemia related to ondansetron use was an underrecognized phenomenon due to the confounders of chemotherapy toxicity and vomiting-induced shifts in K. We therefore conducted a prospective study of patients receiving chemotherapy with ondansetron to determine (1) if renal potassium wasting is a common phenomenon in pediatric oncology patients receiving ondansetron, and (2), if prevalent, which factors increase the probability of potassium wasting in pediatric oncology patients, particularly in those developing clinically apparent hypokalemia.

## 2. Materials and Methods

**Figure 1:**
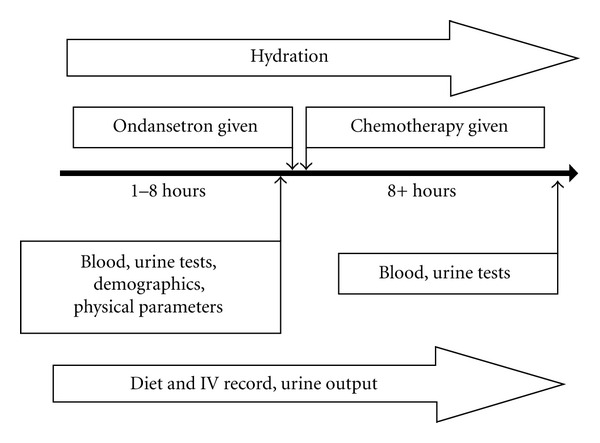
Protocol timeline.

Based on the previous published case report, we designed a prospective cohort study to determine if hypokalemia associated with ondansetron use was an under-recognized complication (Figure 1). The University of AB, Canada, Health Research Ethics Board Biomedical Panel approved the study protocol. Patients were recruited sequentially and consented into the study from the outpatient Pediatric Oncology clinic at the Stollery Children's Hospital in Edmonton, Alberta from January 2008 to August 2008. Patients were selected to participate who required administration of chemotherapy where planned prophylactic administration of ondansetron was expected. Chemotherapy regimens were therefore limited to intravenous cyclophosphamide, cisplatin, ifosfamide, and/or high-dose methotrexate therapy. To remove any influence of high aldosterone states related to intravascular volume depletion and subsequent promotion of renal potassium wasting, all patients recruited into the study received hydration prior to the administration of ondansetron and chemotherapy. Patients were excluded from the study if they were unable to voluntarily provide a urine specimen, had protracted vomiting refractory to anti-emetic therapy at the time of study entry, had received ondansetron within 48 hours of entering the study, or were taking potassium supplements for any reason. No patient had primary renal or adrenal malignancy, noted malignancy involvement of the kidneys, or diuretic use on entering the study (Table 1). 

On admission, demographics including age, gender, height, weight, diagnosis, and chemotherapy protocol were collected. In order to determine sodium and potassium balance for each patient throughout the pre- and posttherapy study period, a dietary history and IV fluid record were obtained and sodium and potassium intake and output were calculated [9]. Before receiving the dose of ondansetron, serum samples were obtained to determine plasma osmolality, potassium, sodium, aldosterone, and creatinine levels. Urine samples were obtained within a few hours of the serum samples and before ondansetron administration. Urine was collected for urinalysis, osmolality, potassium, sodium, and creatinine levels. Urine volume was measured preceding ondansetron administration for 1–8 hours, as well as at least 8 hours after ondansetron administration. IV hydration was administered to ensure euvolemia was achieved prior to administration of high-dose methotrexate, cyclophosphamide, ifosphamide, or cisplatin. Patients who showed signs of intravascular volume depletion prior to chemotherapy administration were permitted additional bolus intravenous normal saline to correct their volume deficit, at the discretion of the attending oncologist. Each patient received at least one dose of ondansetron during the study period, either orally or parentally according to the manufacturer's product monograph [3].

Four to eight hours after ondansetron therapy, serum samples were collected to remeasure plasma osmolality, potassium, sodium, aldosterone, and creatinine levels. Similarly, urine was collected for urinalysis, osmolality, potassium, sodium, and creatinine.

### 2.1. Primary Outcomes

 Primary outcome was change in TTKG. The TTKG measures the extent of potassium secretion in the distal portion of the tubule. It is calculated using the following formula:

(1) TTKG=(K+Urine  ×  OsmolalityPlasma  )(K+Plasma×OsmolalityUrine).

The validity of this measurement relies on three assumptions: (1) few solutes are reabsorbed in the medullary collecting duct (MCD), (2) potassium is neither secreted nor reabsorbed in the MCD, and (3) the osmolality of the fluid in the terminal cortical collecting duct is isoosmolar to plasma [10]. The TTKG is therefore only valid when Osmolality_Urine_ ≥ Osmolality_Plasma_ and urine sodium >25. 

### 2.2. Secondary Outcomes

Secondary outcomes were (1) change in the plasma potassium, (2) change in sodium balance, and (3) change in potassium balance throughout the study interval. Balance studies were undertaken should the participant's urine be hypoosmolar relative to plasma. In short, a dietary history was used to estimate sodium and potassium intake, in conjunction with IV fluid records. Output was assessed using urine volumes and urinary chemistry measurements to calculate total sodium and potassium excreted. Balance calculations were done in both the pre- and postondansetron phase and were normalized to the time frame of measurement and the weight of the patient to allow comparison. No patients were noted to have diarrhea or emesis throughout the course of the observation and all patients had normal renal function.

## 3. Statistical Analysis

We decided that a clinically important treatment effect of ondansetron on the renal tubule would be a doubling of the TTKG and calculating the sample size based on published data [5]. In that report, a single patient was observed to have a reproducible changes in the TTKG with ondansetron exposure. The values from those clinical exposures were used to estimate a mean TTKG before (6.2 ± 2.1) and after ondansetron (11.6 ± 2.4). We therefore used a minimum expected difference of 5.4 with a standard deviation of 2.3. The sample size estimate for this study, using *α* = 0.05, a power of 90%, and assuming a parametric distribution of data, was 7.6 patients. We planned recruitment of 12 patients to insure adequate sampling. Data was assessed for normality using a one-sample Kolmogorov-Smirnov Test using *P* < 0.05 as significance to reject the null hypothesis that the distribution of each variable was nonparametric. All variables were assessed as parametric. As each patient acted as a pretherapy control, variables were subsequently analyzed using a paired two-tailed Student *t*-test, with statistical significance determined by *P* < 0.05. 

## 4. Results 

Twelve patients participated in the study (Table 1). Patient 11 was excluded from the analysis due to inability to collect the required urine samples. Patient 1 developed protracted vomiting during the preondansetron phase and was subsequently excluded. Ten patients therefore completed the study. The patients that completed the study received 2.8 ± 2.4 cc/kg/hr hydration for 7.4 ± 7.2 hours before ondansetron and received 4.8 ± 5.4 cc/kg/hr (*P* = 0.30) for 16.3 ± 9.0 hours (*P* = 0.02) after hydration. Five patients developed a urine osmolality lower than plasma osmolality in the postondansetron phase prohibiting the calculation of TTKG. These patients were excluded from the analysis based on TTKG, but were included in the analysis of potassium and sodium balance before and after ondansetron. Patient 9 had elevated aldosterone levels in the postondansetron phase in the absence of hyperkalemia or acidosis (data not shown). This patient did not have stool loss during the observation period, but was treated with a bowel enema prior to admission, suggesting an explanation for the decreased intravascular volume and elevated aldosterone despite seemingly adequate prechemotherapy hydration. This patient was included in the analysis.

Of the patients that completed the study, no patient developed hypokalemia (Table 2). Assessment of TTKG before and after ondansetron did not demonstrate a significant difference. Patients did develop lower urine osmolality (*P* ≤ 0.05) after hydration and ondansetron therapy. After ondansetron, patients also developed an appropriately lower urinary potassium concentration (*P* = 0.02) and higher urinary sodium excretion (*P* = 0.03). Plasma aldosterone levels trended lower post ondansetron, but were not statistically significant. Evaluation of sodium and potassium output also confirmed that urinary sodium was higher (*P* ≤ 0.05) and urinary potassium was lower (*P* = 0.02) in the after ondansetron phase. However, balance studies did not show appreciable differences before and after ondansetron in these electrolytes.

## 5. Discussion

We conducted a prospective cohort study of patients receiving chemotherapy to determine if renal potassium wasting is a common phenomenon related to ondansetron use. We have demonstrated that when intravascular volume contraction and preexisting tubulopathy predisposing to hypokalemia are controlled for, ondansetron does not appear associated with excessive renal potassium secretion.

Renal potassium handling is, for the most part, influenced by two factors. First, renal tubular flow is important in determining the quantity of potassium in the renal tubule; the faster the flow, the more likely the potassium concentration in the tubule will be diluted. Maintaining a low tubular potassium concentration facilitates the secretion of potassium by maintaining large gradients favoring secretion into the lumen. Low tubular flow states exist in dehydration or with the use of angiotensin-converting enzyme inhibitors and can lead to inability to secrete potassium and hyperkalemia [11]. Secondly, the renal tubule needs to establish both an electrical and a chemical gradients in the distal nephron to facilitate tubular potassium secretion. As already noted, significant factors determining the magnitude of the gradient are both the amount of sodium delivery to the distal nephron, and the presence of aldosterone which facilitates both apical sodium entry into the cell and potassium efflux from the cell into the urine [12].

Our experimental design intentionally exploited a hydration phase of therapy to minimize the effect of aldosterone on the measurement of potassium in the urine. We note that the aldosterone levels did not decrease significantly throughout the study. We believe this is because of two reasons; first, due to the small sample size, we included patient 9 who actually experienced an increase of aldosterone during the study. On review of the chart, we could find no evidence of volume loss through bleeding, gastrointestinal, or other sources to explain a physiologic increase in aldosterone. Furthermore, there was no evidence that the primary disease was affecting adrenal function to explain autonomous mineralocorticoid production. The second reason for the observed nonstatistical difference in aldosterone is because our lab is unable to report plasma aldosterone levels below 69 pmol/L. For the purpose of the analysis, any value reported as “<69” was analyzed as 69 pmol/L. However, clinically significant volume status changes occurred through the period of observation, supported by the significant drop in urine osmolality. Although antidiuretic hormone (ADH) was not measured, the urine osmolality provides an indirect measurement that suggests ADH activity was abolished after the hydration phase [13]. This led to the observation that the primary outcome measure of TTKG could not be used in half of the studied patients.

Urinary sodium was noted to increase throughout the studyand is likely the result of hydration with salt-containing solutions. This is supported by the sodium balance studies that showed significant increases in sodium intake in the latter half of the study, but no change in the net sodium balance. This suggests that the patients were merely excreting what they were given as hydration therapy. We also note that the urine potassium concentration decreased in our patients after ondansetron administration. Interestingly, urine potassium concentration decreased by the same factor as the urine osmolality. This is consistent with the balance studies that show no effect on overall potassium balance and infer the concentration change is not due to altered tubular potassium handling, but instead decreased tubular water reabsorption after adequate hydration.

We previously described a pediatric oncology patient who reliably developed hypokalemia when exposed to ondansetron and reviewed in vitro data that supports a mechanism for ondansetron promoting renal potassium wasting [5]. While we did not observe a similar effect in our prospective cohort study, we note that there are variables related to the initial patient's presentation, which may have facilitated an ondansetron effect. First, the patient we described had a history of prolonged nausea and poor intake, which suggests that the patient may have been chronically nutritionally and total body potassium deplete. In the current study, we excluded patients who had evidence of protracted vomiting and poor nutritional intake. The patient we reported previously showed resistance to potassium replacement therapy initially, suggesting that as nutrients were provided, potassium was shifting intracellularly, potentially under the influence of carbohydrate-induced insulin generation [14]. This may have created an environment that allowed clinically relevant hypokalemia to develop as the renal potassium wasting documented likely occurred in the setting of deplete intracellular stores, preventing an extracellular shift of potassium and allowing plasma potassium levels to fall. All of the patients involved in the study had reasonable oral potassium intake entering into the study, and the nutritional records support that oral intake was adequate during the course of the study. Second, our patient cohort showed a reduction in urine potassium after ondansetron, which did not affect potassium balance. The trend toward the reduction of aldosterone, albeit not statistically significant, did occur with aggressive hydration. Our initial patient had adequate hydration and had no detectable aldosterone at the beginning of our observation, suggesting the baseline state of potassium secretion in the renal tubule in our cohort was different. We also note that although not statistically significant, there was a trend to increase potassium intake via IV administration during the postondansetron phase, as a result of potassium containing IV solutions administered in greater quantity. This may have prevented the development of clinically relevant hypokalemia.

In our study, we allowed the route of administration of ondansetron to be left to the discretion of the attending physician. Several pharmacokinetic parameters were considered in this discussion [16, 17]. Pharmacokinetic data suggests that oral absorption of ondansetron is 100%, although bioavailability is reduced as low as 50% in the general population due to first pass metabolism in the liver. However, in oncology populations, due to induction of hepatic metabolism, bioavailability increases to 85%, suggesting that in the oncology population oral and IV dosing regimens are comparable. Additionally, all ondansetron dosing was on an 8-hour dosing regimen, dosed as needed. The half-life of ondansetron is approximately 3 hours, supporting an observation period of less than 12 hours to see the effect of the drug. Similarly, we did not extend our evaluation beyond 12 hours of monitoring as the likelihood of seeing drug effect would be minimal beyond this time frame. 

Our study was constructed to demonstrate whether ondansetron had a durable association with renal K wasting. We do note that out study does have limitations. First, our study is a small cohort and of a short duration. There are many confounding medications administered in oncology therapy, and we are unable to determine whether specific drug combinations in association with ondansetron promote K wasting in the urine. Similarly, while none of our patients had preexisting tubular dysfunction, we are unable to determine whether preexisting tubular dysfunction is a requirement to induce K wasting when dosing ondansetron. However, our population is quite heterogeneous and does demonstrate that within the scope of pediatric oncology practice, clinically significant hypokalemia was not associated with ondansetron administration. Secondly, we constructed our study with the intention of applying stringent statistical parameters, using a power of 90% in the sample size calculation, which is more stringent than standard. In determining the sample size to be 7 patients, we recruited 12 in anticipation of patient dropout or the possibility of missing data. Based on our observations, only five patients were able to undergo analysis of TTKG. Although this suggests that the study is underpowered based on the primary outcome, we also appreciate that had a standard power of 80% been used, we would have required only 5 patients. However, we acknowledge that the samples size is small and that in a negative study the possibility of failing to detect a significant difference is a limitation of the study. 

We maintain that ondansetron does not appear to be associated with clinically significant hypokalemia. Although we observed clinically apparent hypokalemia in one patient, when controlling for hydration, adequate nutritional intake, and receiving adequate potassium replacement, our cohort did not demonstrate a negative potassium balance or the development of clinically relevant hypokalemia. Consideration should be given to ensure maintenance potassium replacement is offered during ondansetron administration for chemotherapy-induced nausea prophylaxis early in the course of treatment, particularly in patients with poor hydration status or poor nutritional status.

## Figures and Tables

**Table 1:** Patient characteristics, diagnosis, and medication history.

Pt	Age (years)	Gender	Height (cm)	Weight (kg)	Diagnosis	Chemotherapy protocol and additional medications	Medications received 24 hr prior to observation	Medications received during preondansetron observation period	Medications received during postondansetron observation period
1	11.5	F	146	34.5	Before B-cell ALL	AALL 0232 Cyclophosphamide 1000 mg/m^2^ × 1 Cytarabine 75 mg/m^2^/d × 13 6-Mercaptopurine 60 mg/m^2^/d × 13 Vincristine 1.5 mg/m^2^ × 1 PEG Asparaginase 6000 IU/m^2^ × 1 IT Methotrexate 15 mg/m^2^ × 6 IT Cytarabine 70 mg/m^2^ Prednisone 60 mg/m^2^/d × 28 days Daunorubicin 25 mg/m^2^/d × 4 Other: Trimethoprim/sulfamethoxazole 160 mg (TMP) po BID × 3 days Colace 100 mg po BID Senokot 1 tablet po QHS	None	None	None

2	16.5	M	181	68.6	Osteogenic sarcoma	COG AOST 0331 Cycle 2, week 9 Methotrexate 12 g/m^2^ Other: Trimethoprim/sulfamethoxazole 160 mg (TMP) q AM and 80 mg (TMP) po qPM × 3 days Colace 100 mg po BID	None	None	Decadron 10 mg po × 1, 5 mg po × 1, Methotrexate 20 g IV × 1

3	16.8	M	167	52.2	Extragonadal pure seminoma, stage 3	CCG-8891/COG 9048 Cisplatin 20 mg/m^2^ × 5 days Etoposide 100 mg/m^2^ × 5 d Bleomycin 15 U/m^2^ × 1 Other: Trimethoprim/sulfamethoxazole 160 mg (TMP) q AM and 80 mg (TMP) po qPM × 3 days Colace 100 mg po BID Senna^2^ tablets po BID Lactulose 30 mL BID PRN Codeine 16 mg q4h PRN	None	None	Ondansetron 8 mg po × 4 Decadron 10 mg × 2, 5 mg × 3 Nabilone 1 mg × 3 Colace 100 mg po × 1 Bleomycin 24 mg IV × 1 Etoposide 160 IV × 2, Cisplatin 32 mg × 2

4	16.5	F	164	53.2	Nodular sclerosing Hodgkin's lymphoma, stage 1A	COG AOHD 0431 Cycle 2 Doxorubicin 25 mg/m^2^/day 1, 2 Vincristine 1.4 mg/m^2^ day 1, 8 Prednisone 40 mg/m^2^ day 1–7 Cyclophosphamide 600 mg/m^2^ day 1–7 G-CSF 5 mcg/kg d Other: Trimethoprim/sulfamethoxazole 160 mg (TMP) po BID × 3 days	Cascara 5 mL po × 1 Magnesium hydroxide 25 mL po × 1	None	Ondansetron 8 mg × 3 Ranitidine 150 mg po × 1 Prednisone 30 mg × 1 Dimenhydrinate 50 mg po × 1

5	16.6	F	177	55.6	Nodular sclerosing Hodgkin's lymphoma, stage 3	AHOD-0031 Cycle 2 with DECA Doxorubicin 25 mg/m^2^/d × 2 Bleomycin 5 U/m^2^/d day 1, 10 U/m^2^/d day 8 Vincristine 1.4 mg/m^2^/d day 1, 8 Etoposide 100 mg/m^2^/d Prednisone 40 mg/m^2^/d Cyclophosphamide 800 mg/m^2^ × 1 Dexamethasone 10 mg/m^2^ day 1, 2 Cytarabine 3000 mg/m^2^ day 1, 2 Cisplatin 90 mg/m^2^ IV day 1 GCSF 5 mcg/kg/d Other: Trimethoprim/Sulfamethoxazole 160 mg (TMP) po BID × 3 days	Prednisone 35 mg po × 1	None	Ondansetron 8 mg × 1 Dimenhydrinate 50 mg IV × 2 Doxorubicin 42.5 mg Bleomycin 8.5 U at Vincristine 2.4 mg at Etoposide 213 mg Cyclophosphamide 1360 mg

6	7.5	M	118	23.0	Medulloblastoma	COG CNS 0331 Regimen B Vincristine 1.5 mg/m^2^ day 1, 8 Cyclophosphamide 1000 mg/m^2^ day 1, 2 MESNA 360 mg/m^2^ × 3 doses with each Cyclophosphamide Other: Trimethoprim/sulfamethoxazole 80 mg (TMP) po BID × 3 days	None	None	Decadron 4 mg IV × 1, Ondansetron 4 mg IV × 1 Vincristine 1.4 mg IV Mesna 325 mg IV, Cyclophosphamide 900 mg IV

7	12.5	M	156	61.7	Before B-cell ALL	AALL 0232 Cyclophosphamide 1000 mg/m^2^ × 1 Cytarabine 75 mg/m^2^/d × 13 6-Mercaptopurine 60 mg/m^2^/d × 13 Vincristine 1.5 mg/m^2^ × 1 PEG Asparaginase 6000 IU/m^2^ × 1 IT Methotrexate 15 mg/m^2^ × 6 IT Cytarabine 70 mg/m^2^ Prednisone 60 mg/m^2^/d × 28 days Daunorubicin 25 mg/m^2^/d × 4 Other: Trimethoprim/sulfamethoxazole 160 mg (TMP) po BID × 3 days Domperidone 10 mg po QID Citalopram 10 mg po daily Omeprazole 20 mg po daily	Dimenhydrinate 50 mg IV × 1	None	Cyclophosphamide 800 mg IV × 1 Domperidone 10 mg × 1 6-Mercaptopurine 40 mg × 1

8	6.0	M	112	19.8	Biphenotypic leukemia	Institutional Protocol Ifosfamide 2.5 g/m^2^/d × 3 Etoposide 100 mg /m^2^/d × 3 Carboplatin 635 mg/m^2^ day 3 GCSF 5 mcg/kg/d Other: Trimethoprim/sulfamethoxazole 80 mg (TMP) po BID × 3 days Dimenhydrinate 25 mg po q6h PRN	Decadron 4 mg IV × 1 Voriconazole 100 mg po bid	None	Ondansetron 4 mg × 1 Decadron 2 mg IV × 1

9	14.8	M	171	88.2	Before B-cell ALL	COG AALL 0232 Cyclophosphamide 1000 mg/m^2^ × 1 Cytarabine 75 mg/m^2^/d × 13 6-Mercaptopurine 60 mg/m^2^/d × 13 Vincristine 1.5 mg/m^2^ × 1 PEG Asparaginase 6000 IU/m^2^ × 1 IT Methotrexate 15 mg/m^2^ × 6 IT Cytarabine 70 mg/m^2^ Prednisone 60 mg/m^2^/d × 28 days Daunorubicin 25 mg/m^2^/d × 4 Other: Trimethoprim/Sulfamethoxazole 160 mg (TMP) po BID × 3 days	None	None	Cytarabine 150 mg IV Cyclophosphamide 2 g IV Ondansetron 8 mg IV × 1

10	13	F	139	33.5	Before B-cell ALL relapse	Institutional Protocol Relapse 001 Maintenance Cytarabine 2.9 mg/m^2^/d Etoposide 290 mg/m^2^/d Vincristine 0.7 mg/m^2^/d Dexamethasone 6 mg/m^2^/d Methotrexate po 200 mg/m^2^/d 6-Mercaptopurine 75 mg/m^2^/d Cyclophosphamide 1100 mg/m^2^/d × 1 Methotrexate IV 1000 mg/m^2^/d × 1 Leucovorin 10 mg/m^2^/d IT Methotrexate 65 mg/m^2^/d IT Cytarabine 25 mg/m^2^/d Other: Trimethoprim/Sulfamethoxazole 80 mg (TMP) po BID × 3 days	None	None	Methotrexate 1100 mg IV × 1

11	10.8	F	154	43.8	Osteogenic sarcoma	AOST 0331, Cycle 2 week 9 Methotrexate 12 g/m^2^ Other: Trimethoprim/Sulfamethoxazole 160 mg (TMP) po BID × 3 days Colace 100 mg po daily PRN	Nabilone 0.3 mg po bid	None	Ondansetron 8 mg IV × 3, Decadron 8 mg IV/po prechemo, Decadron 4 mg IV/po q6h × 3, Nabilone 0.5 mg po BID, Diphenhydramine 50 mg IV

12	16.8	M	173	57.5	Ewing's sarcoma, stage 4	AEWS0031 week 1 Vincristine 1.5 g/m^2^ day 1 Ifosfamide 3 g/m^2^ day 1, 2, 3 Doxorubicin 20 mg/m^2^ day 1, 2, 3 Etoposide 150 mg/m^2^ day 1, 2, 3 Other: Trimethoprim/Sulfamethoxazole 160 mg (TMP) po BID × 3 days	Dimenhydrinate 25 mg po × 1	None	Ondansetron 8 mg × 2 Lactulose 10 mL po × 2 Decadron 10 mg IV × 1 Decadron 5 mg IV × 3 Furosemide 10 mg IV × 2, Nabilone 0.5–1 mg po × 2 Tylenol 975 mg po × 1 Dimenhydrinate 25 iv q4h × 4

**Table 2:** Comparative values of TTKG, Na and K balance before and after ondansetron show no effect on renal K wasting.

	*n*	Before ondansetron	After ondansetron	*P*
PK (mmol/L)	10	3.9 ± 0.3	3.9 ± 0.2	0.774
UK (mmol/L)	10	50.7 ± 22.7	24.7 ± 22.8	0.019
POsm (mmol/kg)	10	287.2 ± 4.2	283.8 ± 18.0	0.573
UOsm (mmol/kg)	10	761.4 ± 183.3	356.7 ± 123.4	0.001
PAld (pmol/L)	10	323.4 ± 484.8	153.9 ± 268.5	0.353
TTKG	5*	5.1 ± 1.9	4.7 ± 2.8	0.463
eGFR (ml/min/1.73 m^2^)	10	148.9 ± 17.9	166.8 ± 46.8	0.207
PNa (mmol/L)	10	138.6 ± 1.8	133.9 ± 18.0	0.426
FENa (%)	10	0.8 ± 0.6	3.1 ± 2.54	0.025

Balance studies				

Na intake (mmol)	10	2840.0 ± 2034.1	8032.8 ± 7257.5	0.080
Na output (mmol)	10	50.7 ± 22.7	113.8 ± 41.6	0.002
K intake (mmol)	10	1459.7 ± 1306.1	2274.0 ± 1693.0	0.347
K output (mmol)	10	50.7 ± 22.7	24.8 ± 22.8	0.019
Net Na balance (mmol/kg/hr observation)	10	0.15 ± 0.39	0.06 ± 0.11	0.520
Net K balance (mmol/kg/hr observation)	10	0.11 ± 0.07	0.39 ± 0.82	0.305

Values are presented as mean ± standard deviation.

Student's *t*-test, paired, two-tailed, significance *P* < 0.05.

Net balance = (intake − output)/weight (kg)/time (hr) of observation period.

PK: plasma potassium, mmol/L; UK: urine K, mmol/L; Posm: plasma osmolality, mmol/kg; Pald: plasma aldosterone, pmol/L (lower limit of detection 69 pmol/L); TTKG: transtubular potassium gradient; eGFR: estimated glomerular filtration rate (Schwartz [15]); Pna: plasma sodium, mmol/L; FENa: fractional excretion of sodium (%).

*5 subjects were not included in this analysis as they developed hypotonic urine relative to blood, invalidating the TTKG assumptions.
